# Acute necrotizing encephalopathy associated with COVID-19 in a pediatric patient and a systematic review

**DOI:** 10.70962/jhi.20250101

**Published:** 2026-02-04

**Authors:** Kanako Takeuchi, Yuichi Tateishi, Kosuke Ashihara, Takanori Utsumi, Yoshiyuki Kobayashi, Takaki Asano, Satoshi Okada

**Affiliations:** 1 Department of Pediatrics, Graduate School of Biomedical and Health Sciences, Hiroshima University, Hiroshima, Japan

## Abstract

Coronavirus disease 2019 (COVID-19), caused by SARS-CoV-2, can lead to neurological complications such as acute necrotizing encephalopathy (ANE). However, the pathogenesis of ANE associated with COVID-19 remains unclear. We report a case of ANE associated with COVID-19 and then conducted a literature review using PubMed for cases reported up to June 30, 2024. Clinical data from 74 patients were analyzed. Among those with severe sequelae (*n* = 28), 39.2% (*n* = 11) had hemorrhagic findings on CT or MRI, significantly more than in the mild sequelae group (10.8% [*n* = 5], P = 0.04). 16 patients underwent genetic testing for *RANBP2* mutations, of whom four (25%, *n* = 16) tested positive. These findings suggest that imaging evidence of hemorrhage may be a poor prognostic factor and that *RANBP2* mutations could contribute to disease susceptibility. Further genetic studies involving larger cohorts are needed to better understand the mechanisms of ANE associated with COVID-19 and improve outcomes for affected patients.

## Teaser

Hemorrhage detected on imaging may predict poor outcomes in acute necrotizing encephalopathy associated with COVID-19. RANBP2 mutations have been identified in some cases, suggesting a genetic contribution to disease susceptibility and highlighting the need for further research.

## Introduction

Coronavirus disease 2019 (COVID-19) has been pandemic worldwide for several years, and started in China in December 2019 with cases of pneumonia of unknown origin. By March 2023, millions of people had died worldwide. The most frequent symptoms of COVID-19 are fever, cough, and dyspnea, and COVID-19 is also associated with neurological disorders such as cerebrovascular disease, encephalitis, meningitis, anosmia and lethargy, Guillain–Barré syndrome, and acute encephalopathy (1, 2).

Acute encephalopathy is known as a heterogeneous clinical and genetic syndrome, demonstrating various forms of disease types depending on their broad clinical manifestations. Among the disease types, acute necrotizing encephalopathy (ANE) is a severe subtype of acute encephalitis/encephalopathy proposed by Mizuguchi et al. in 1995 (3). They have proposed diagnosing the ANE based on findings from imaging studies and cerebrospinal fluid (CSF) analysis. Compared with other forms of acute encephalopathy, ANE is associated with a poorer prognosis; the mortality rate of ANE is estimated to be ∼30%, and neurological sequelae are observed in many survivors (4, 5, 6). At present, although its etiology and pathogenesis remain unclear, ANE has been associated with a variety of viral infections, including influenza A, influenza B, novel influenza A (H1N1), parainfluenza, varicella zoster virus, human herpesvirus (HHV) 6 and 7 (HHV-6 and HHV-7), enterovirus, rotavirus, herpes simplex virus, rubella, coxsackievirus A9, and measles (7). Among these viruses, the influenza virus is often detected in patients with ANE (7). Cytokines, such as tumor necrosis factor-a, interleukin (IL)-1b, IL-2, IL-6, IL-10, and IL-15, play important roles in ANE. They cause a cytokine storm resulting from overactivation of the innate immune response, leading to blood‒brain barrier dysfunction, increased vascular permeability, and subsequently brain edema (8, 9, 10). Furthermore, it has been reported that elevated serum aminotransferase and CSF protein levels, along with the presence of hemorrhage and localized tissue loss on magnetic resonance imaging (MRI), predict poor prognosis (11, 12).

Most ANE cases are sporadic and not recurrent, whereas some cases are familial and recurrent, involving heterozygous missense variants in the RAN-binding protein 2 (*RANBP2*) gene (13). Patients with the *RANBP2* variant have the same clinical course and various radiological findings, such as lesions occurring outside of the brainstem and thalamus. These findings were observed with incomplete penetrance. They have a wide onset of age, and 30% of patients are reported to achieve neurological recovery (14). Recently, severe acute respiratory syndrome coronavirus 2 (SARS-CoV-2) has also been associated with the onset of ANE (15). In contrast, neither the pathogenesis and clinical features of ANE associated with COVID-19 nor the involvement of the *RANBP2* gene are clear.

We report a case of ANE associated with COVID-19 and a review of all the literature from May 2020 through June 2024 on ANE associated with COVID-19 to address the clinical and genetic features of this disease.

## Results

### Clinical report

**Figure 1.**
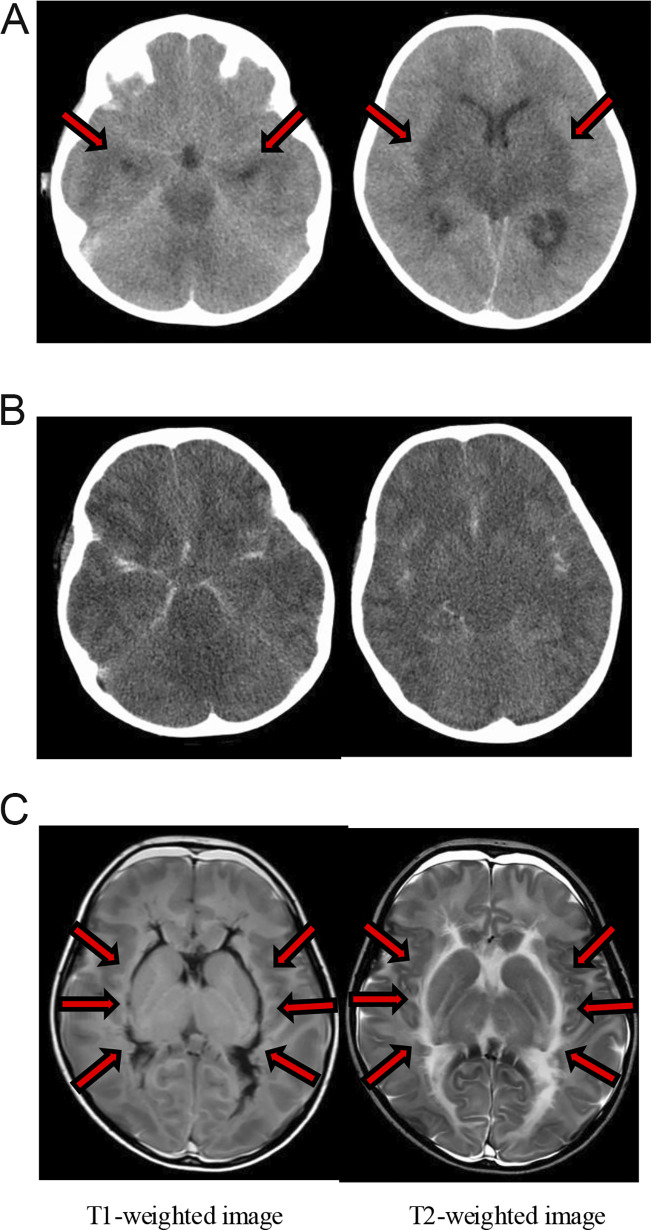
**Brain CT and MRI of our patient. (A)** Initial brain CT scan at disease onset showing hypoattenuation in the cerebral cortex, basal ganglia, thalamus, brainstem, and cerebellum (arrows), along with marked cerebral edema. **(B)** Follow-up brain CT on hospital day 28 demonstrating progression of hypoattenuation throughout the cerebral white matter. **(C)** Brain MRI on day 93 showing findings suggestive of liquefactive necrosis. T1-weighted image showed hypointensity, and T2-weighted image showed hyperintensity in the periventricular and external capsule regions (arrows), indicating parenchymal liquefactive necrosis.

**Figure 2.**
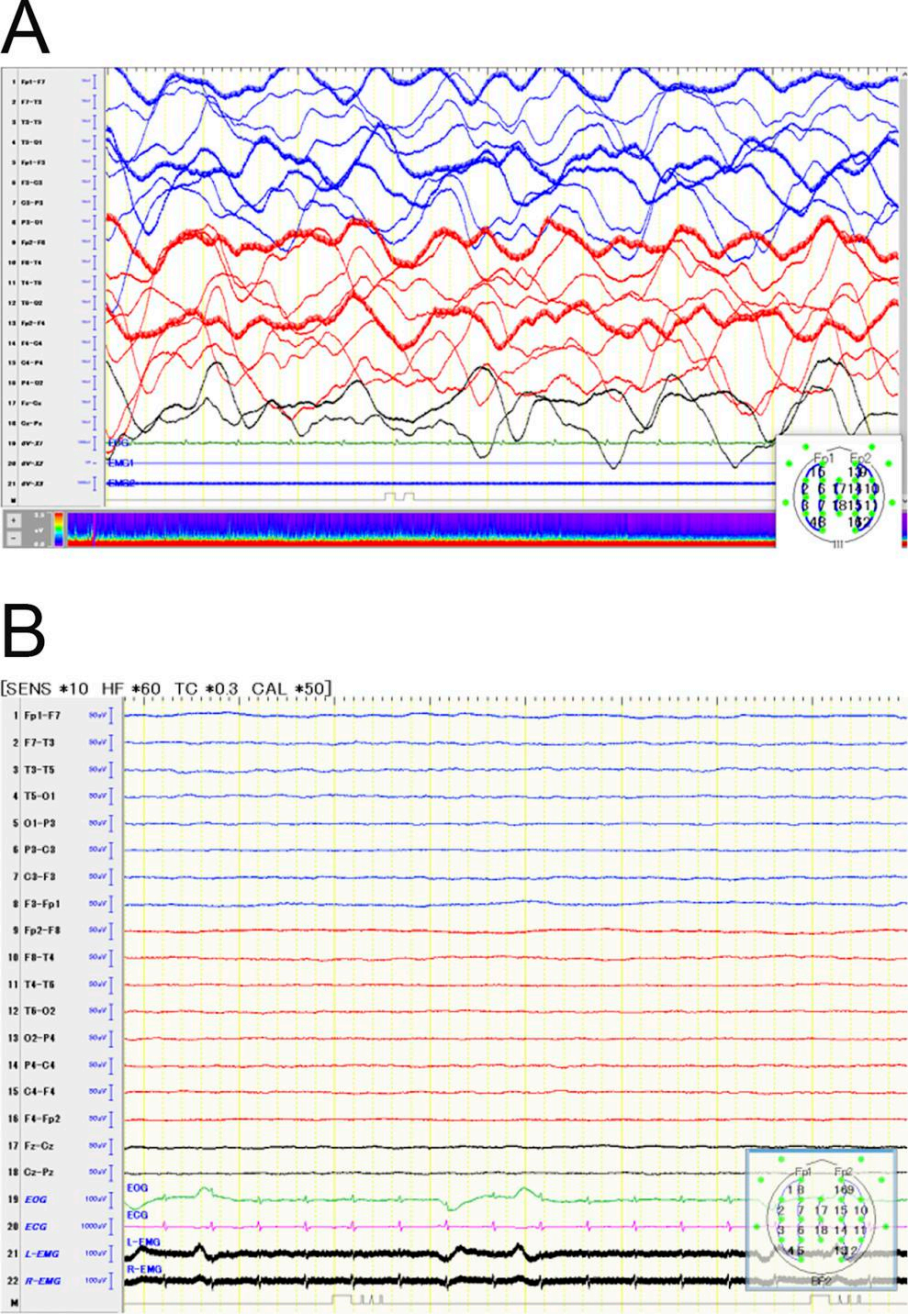
**EEG of our patient.** In a bipolar longitudinal montage, the red curves depict the right hemisphere, the blue curves depict the left hemisphere, and the black curves depict the midline. **(A)** The EEG was recorded at the onset of the disease and shows persistent widespread high-voltage slow waves. **(B)** The EEG showed marked diffuse low-voltage activity, suggesting a marked decline in cerebral function.

In August 2022, the patient was a 3-year-old girl who had a fever on day 1 and was subsequently admitted to the hospital with a decreased level of consciousness on day 2. She had no past history and COVID-19 vaccination. The PCR test for SARS-CoV-2 was positive in the throat swab. At that time, the Omicron variant was predominant in Japan, although the specific SARS-CoV-2 strain in this patient was not investigated. The main datasets for her blood examination and CSF test are presented in Table 1. A brain computed tomography (CT) scan revealed hypoabsorption areas in the cerebral cortex, basal ganglia, thalamus, brainstem, and cerebellum and marked cerebral edema (Fig. 1 A). An electroencephalogram (EEG) revealed persistent widespread high-voltage slow waves (Fig. 2 A). On the basis of the clinical course and laboratory findings, we diagnosed the patient with ANE related to SARS-CoV-2 infection. In the intensive care unit, circulatory support was provided with vasopressors. As treatment for acute encephalopathy, she received methylprednisolone pulse therapy, intravenous immunoglobulin, edaravone, mannitol, vitamin cocktail therapy, and L-carnitine. However, her level of consciousness did not improve. On day 3, her pupils were bilaterally dilated, and the light reflex was absent. Additionally, her EEG showed marked diffuse low-voltage activity (Fig. 2 B), indicating a condition consistent with brain death. On day 18, a tracheotomy was performed. A follow-up brain CT on day 28 showed progression of hypoattenuation in the cerebral cortex, basal ganglia, thalamus, brainstem, and cerebellum (Fig. 1 B). On day 42, she presented with hypotension and polyuria. Blood examination revealed hypothyroidism and adrenal insufficiency. She was diagnosed with central diabetes insipidus, hypothyroidism, and adrenal insufficiency secondary to hypopituitarism. Hormone replacement therapy was initiated with desmopressin, levothyroxine sodium, and hydrocortisone. On day 93, brain MRI revealed hyperintensity on T2-weighted images and hypointensity on T2 FLAIR in the periventricular and external capsule regions, indicating parenchymal liquefactive necrosis (Fig. 1 C). Finally, she was discharged on day 103. She remains in a persistent unresponsive state with no brainstem reflexes and continues to receive respiratory support and hormone replacement therapy while being cared for at home 3 years after onset. Although we underwent genetic testing later, we found no pathological variants associated with inborn errors of immunity or neurological disease.

**Table 1.** Laboratory data of our patient at the onset of the disease

Samples	Patient value (reference ranges)
White blood cell count	6,880 (/μl) (4,200–18,000)
Lymphocytes	3.7 (×10^3^/μl) (2.0–8.0)
Neutrophils	2.9 (×10^3^/μl) (1.5–8.5)
Hemoglobin	10.0 (g/dl) (11.1–14.2)
Platelet count	1.0 × 10^6^ (/μl) (18.0–58.0 × 10^4^)
Blood urea nitrogen	22.7 (mg/dl) (5.5–19.3)
Creatinine	0.74 (mg/dl) (0.2–0.39)
Aspartate aminotransferase	540 (U/L) (24–44)
Alamine aminotransferase	184 (U/L) (9–30)
Lactate dehydrogenase	1,084 (U/L) (190–365)
C-reactive protein	0.33 (mg/dl) (0–0.14)
Ferritin	5,622.3 (ng/ml) (3.6–114)
D-dimer	140.9 (μg/ml) (0.15–1.0)
Procalcitonin	51.3 (ng/ml) (0–0.49)
CSF cell count	7.0 (/μl) (0–8.0)
CSF protein	11.5 (mg/dl) (14.6–20.8)

Values in parentheses indicate the reference ranges for Japanese children in healthy status.

### Literature review

**Figure 3.**
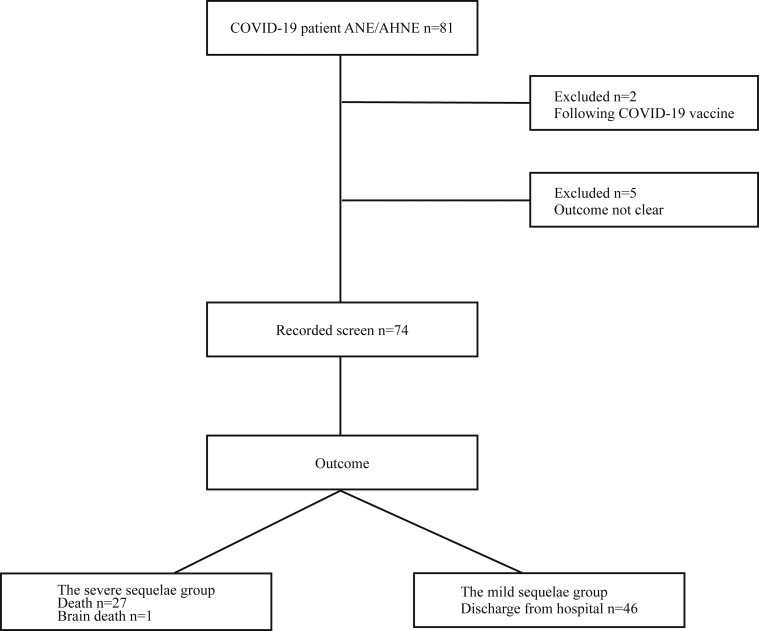
**Patient flow chart.** We divided these 74 patients into two groups: (1) the severe sequelae group, which included patients who died or were in a state of brain death, and (2) the mild sequelae group, which included patients who were discharged with neurological comorbidities.

Among the 319 selected reports published from 2020 to 2024, we analyzed available data from 81 patients with ANE associated with COVID-19 (16, 17, 18, 19, 20, 21, 22, 23, 24, 25, 26, 27, 28, 29, 30, 31, 32, 33, 34, 35, 36, 37, 38, 39, 40, 41, 42, 43, 44, 45, 46, 47, 48, 49, 50, 51, 52, 53, 54, 55, 56, 57, 58, 59, 60, 61, 62, 63). Among the 81 patients, seven were excluded: two patients were diagnosed with ANE after vaccination, and the outcomes of five patients were not described. Overall, we analyzed 74 cases (Fig. 3). Of the 74 cases, 33 were adults and 41 were children. We divided these 74 patients into two groups: (1) the severe sequelae group, which included patients who died or were in a state of brain death, and (2) the mild sequelae group, which included patients who were discharged with neurological comorbidities. The demographics, clinical characteristics, and test results of the 74 patients are summarized in Table S1.

**Figure 4.**
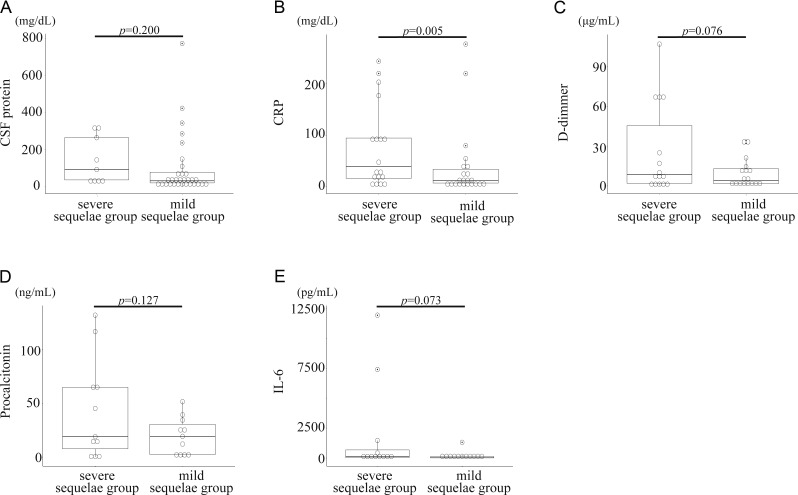
**A comparison of laboratory data. (A and C)** CSF protein and D-dimer tended to be higher in the death or severe sequelae group, but no significant difference was observed (A: P = 0.200, C: P = 0.076). **(B)** Patients in the death or severe sequelae group showed significantly higher CRP levels in blood tests than in the discharge from hospital (P = 0.005). **(D and E)** Due to the limited data, no significant differences were observed for procalcitonin and blood IL-6 levels.

**Figure 5.**
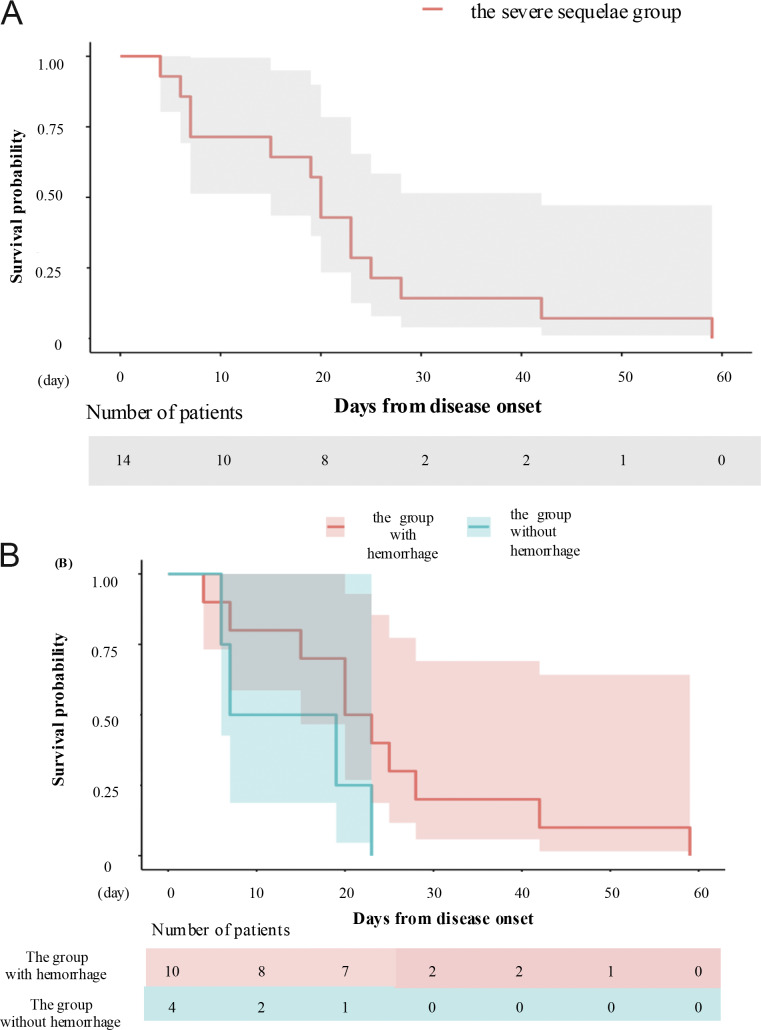
**Kaplan–Meier survival curves in the severe sequelae group. (A)** The Kaplan–Meier curve of the patients in the severe sequelae group (*n* = 14) is shown. The numbers below the Kaplan–Meier curve represent the number of patients who remained alive at each time point after disease onset. Among the patients in the severe group, data were available for 14 individuals, almost all of whom died or progressed to brain death within one month after onset. The shaded area indicates the 95% confidence interval. **(B)** Patients in the severe sequelae group were divided into two subgroups: with hemorrhage (red, *n* = 10) and without hemorrhage (green, *n* = 4). Kaplan–Meier survival curves were plotted for each subgroup. The numbers below the curves indicate the number of patients who remained alive at each time after disease onset. Patients without hemorrhage tended to have shorter survival times than those with hemorrhage, but due to the small sample size, no statistically significant difference was observed between the two groups (P = 0.05). The shaded areas indicate the 95% confidence intervals.

The median age of onset was 11 years (35 days–81 years), with a 50% female ratio. Comparisons between the two groups were made for age, sex ratio, medical history, and the percentage of patients under 15 years of age. The mild sequelae group was significantly younger than the severe sequelae group (P = 0.0238). The other variables did not significantly differ (sex ratio: P = 0.45; medical history: P = 0.60; percentage of patients under 15 years of age: P = 0.24). We extracted partial data from these patients and compiled them in Table 2. In the severe sequelae group (*n* = 28), 11 patients (39.2%) had findings of hemorrhage on brain CT or MRI, and this percentage was significantly greater than that in the mild sequelae group (5 in 46, 10.8%) (P = 0.007) (Table 2). The median duration from disease onset to neurological symptoms tended to be longer in the severe sequelae group. However, due to limited data, a statistically significant analysis could not be performed (Table 2). Patients in the severe sequelae group had higher C-reactive protein (CRP) levels in blood tests compared to those in the mild sequelae group (Table 2 and Fig. 4 C). CSF protein levels also tended to be higher in the severe sequelae group, but the difference was not statistically significant, likely due to the small sample size (Table 2 and Fig. 4 A). Among the data obtained for 14 patients, most patients in the severe sequelae group died or experienced brain death within one month (Fig. 5 A). We compared survival times between the ANE group with hemorrhage and the ANE group without hemorrhage. Although the ANE group without hemorrhage tended to have longer survival times, no statistically significant difference was observed between the two groups, likely due to the limited number of patients (P = 0.05) (Fig. 5 B). In the mild sequelae group, patient prognosis was evaluated using the modified Rankin scale (mRS) score (64). We defined a good prognosis as an mRS score ≦2 and a poor prognosis as an mRS score >2. Overall, nine patients (19.5%) in the discharge group had a good prognosis. The remaining 25 patients (54.3%) had mRS scores >2, exhibited gait and cognitive impairments, and required assistance for daily activities. The discharge status of the remaining 12 patients was unknown. Genetic testing was conducted for 16 patients; four patients (25.0%) had *RANBP2* mutations (Table S1). Three patients had the *RANBP2* p.Thr585Met (T585M) monoallelic mutation. This variant, as well as other pathogenic *RANBP2* variants, has been previously reported in ANE cases following various viral infections such as influenza, HHV-6, respiratory syncytial virus, and parainfluenza virus, supporting its role as a predisposing factor for ANE (14, 65, 66).

**Table 2.** Partial imaging and laboratory data of the patients

​	Severe sequelae group (*n* = 28)	Mild sequelae group (*n* = 46)	P value
**Hemorrhage**	​	​	​
Data available	28	46	​
Number of patients	11/28 (39.2%)	5/46 (10.8%)	0.007
**Time duration from onset to neurological symptoms**	​	​	​
Data available	6	19	​
Time duration (median; day)	8.5	4	0.386
**CSF test**	​	​	​
Data available	9	31	​
CSF protein level (median; mg/dl)	230	78	0.200
**Blood test**	​	​	​
CRP	​	​	​
Data available	18	22	​
CRP level (median; mg/L)	39.6	11.6	0.005
D-dimer	​	​	​
Data available	14	17	​
D-dimer level (median; μg/ml)	8.7	3.7	0.076

## Discussion

ANE is a rare and severe neurological disorder. Intriguingly, ANE is more common in Asia than in other regions, whereas sporadic cases have been reported in North America and Europe (67). ANE has a poorer prognosis than other forms of acute encephalopathy, with an estimated mortality rate of ∼30% (68, 69). The direct effects and sequelae of virus infection lead to serious neurological outcomes and significantly high mortality when the central nervous system is involved (70).

The frequency and characteristics of ANE caused by COVID-19 remain unclear. According to the report published by Li et al. in 2024, 167 (32.8%) of the 509 pediatric patients hospitalized with Omicron variant infection exhibited neurological symptoms. Among these patients, six (3.6%) were diagnosed with ANE, five of whom (83.3%) died (71). Another report indicated that many patients with ANE associated with COVID-19 developed systemic inflammatory response syndrome, leading to multiple organ failure (63). Therefore, multiple organ failure may significantly contribute to the high mortality rate among patients with ANE associated with COVID-19. We hypothesized that ANE associated with COVID-19 has a poorer prognosis than other viruses do and analyzed the clinical features of patients with ANE associated with COVID-19.

Our literature review revealed that almost all patients with ANE presented with a decreased level of consciousness, seizures, and focal neurological deficits. Patients in the severe sequelae group were significantly more likely to have findings of hemorrhage on brain CT or MRI than those in the mild sequelae group. This finding aligns with that of a previous study reporting that findings of hemorrhage on CT and MRI scans are associated with a poor prognosis of ANE caused by other viruses (12). Furthermore, the median CSF protein level was greater in the severe sequelae group than in the mild sequelae group (P = 0.20). However, owing to the severe course of the disease, CSF testing was performed in only half of the patients, which may have contributed to the lack of significant differences. Elevated CSF protein levels have been reported as a poor prognostic factor in previous ANE reports (11, 12), and they may be a poor prognostic factor in patients with ANE associated with COVID-19.

The etiology and pathogenesis of ANE associated with COVID-19 remain unclear, but it has been reported that aberrant cytokine storms caused by overactivation of the innate immune response against SARS-CoV-2 infection play important roles in these patients (72). A systematic review reported that IL-6 and IL-10 levels were significantly elevated in patients with severe COVID-19 and that they could be biomarkers of disease severity (73). In this study, IL-6 levels were elevated in both the deceased and discharged groups (Fig. 4 E). The deceased group included patients with abnormally high blood IL-6 levels, suggesting a possible relationship between blood IL-6 levels and the severity of ANE.

Neilson et al. initially reported heterozygous missense *RANBP2* mutations as a genetic cause for recurrent and familial ANE with incomplete penetrance (13). The pathogenic mechanism of *RANBP2* mutation remains unclear. Some authors report that *RANBP2* may be a critical modulator of neuronal activity, glucose catabolism, and energy homeostasis (74). In contrast, there are familial and recurrent ANE patients without *RANBP2* mutation, demonstrating that the genetic contribution to ANE has not yet been fully elucidated (75). In this study, 16 patients were tested for *RANBP2* mutations, and four patients had positive results (Table S1). Only one patient had a family history or a history of acute encephalopathy, whereas three patients had neither a family history nor a history of acute encephalopathy. Among these patients, three had the *RANBP2* p.Thr585Met (T585M) mutation. This variant is quite rare, as reported in gnomAD v4.1.0, with a minor allele frequency of 0.00000124 (https://gnomad.broadinstitute.org/gene/ENSG00000153201?dataset=gnomad_r4). Moreover, 10 pathogenic mutations in *RANBP2* have been recently reported, with the *RANBP2* T585M mutation accounting for the majority (52, 76). The *RANBP2* mutation may also be related to ANE associated with COVID-19.

Our patient showed no hemorrhage on brain CT, but the prognosis was poor. Because of the acute severe course of the disease, we did not perform detailed examinations. Even among the patients with limited laboratory examinations, we detected high D-dimer levels. The review included patients with high D-dimer levels in the severe sequelae group, and high D-dimer levels may be a poor prognostic factor. We could not detect a significant difference in the D-dimer value between the two groups in this study, which may be due to the limited number of patients with ANE.

This study had several limitations. First, as this was a retrospective study based on previously published literature, it was not possible to collect detailed laboratory data for all case reports. Second, standardized protocols for diagnosis and treatment were not considered, so different treatment strategies may have affected the outcomes. Third, because reports of ANE associated with COVID-19 are still limited, it is difficult to conduct analyses with strict inclusion criteria, and in the present analysis as well, the cases included a wide age range from children to the elderly. In addition, many cases lacked sufficient diagnostic evaluation due to the typically severe clinical course. Therefore, we need to state that a greater accumulation of cases is required to elucidate the full picture of this disease. Finally, ANE associated with COVID-19 may be associated with *RANBP2* gene mutations, and genetic testing, including assessment of *RANBP2*, should be considered when diagnosing ANE.

### Conclusion

We reported the clinical course of a patient with ANE associated with COVID-19 and comprehensively reviewed the clinical characteristics of ANE associated with COVID-19. The hemorrhage findings on imaging are presumed to be a poor prognostic factor from the systemic study of previous reports. Considering the relatively high number of patients with *RANBP2* mutations, further accumulation of cases with genetic studies are necessary to understand the pathophysiology of ANE caused by COVID-19.

## Materials and methods

### Literature search and data extraction

A comprehensive search was conducted in PubMed, using the keywords “COVID-19” and “ANE.” The reference lists of included studies and relevant reviews were manually searched to ensure literature saturation. The search was conducted on June 30, 2024. Insufficient patient data or without a definite SARS-CoV-2 diagnosis were excluded. The following data were extracted: age, sex, medical history, time to onset of first symptoms of ANE, outcome, laboratory data from CSF and blood test, and head imaging scans.

### Statistical analysis

Statistical analyses were performed using R software, version 4.3.1 (R Project for statistical computing). The chi-square test or Fisher’s exact test was used for binary variables. Significance was set at P < 0.05.

### Online supplemental material

Table S1 summarizes the demographics, clinical characteristics, laboratory findings, imaging results, and outcomes of the 74 patients included in this systematic review. ANE was defined as ANE without hemorrhage, whereas acute hemorrhagic necrotizing encephalopathy was defined as ANE with hemorrhage.

## Supplementary Material

Table S1 summarizes the demographics, clinical characteristics, laboratory findings, imaging results, and outcomes of the 74 patients included in this systematic review. ANE was defined as ANE without hemorrhage, whereas acute hemorrhagic necrotizing encephalopathy (AHNE) was defined as ANE with hemorrhage.

## Data Availability

The clinical data of the pediatric patient included in this study are not publicly available due to patient privacy restrictions. These data are available from the corresponding author upon reasonable request with approval from the relevant institutional ethics committee. The data underlying the tables and figures in the systematic review section were derived entirely from previously published studies. All source data are in the public domain and can be accessed through the references cited in the manuscript.
